# Biocompatibility of mineral trioxide aggregate and biodentine as root-end filling materials: an in vivo study

**DOI:** 10.1038/s41598-024-53872-w

**Published:** 2024-02-12

**Authors:** Mohamed Nabeel, Ashraf M. Abu-Seida, Abeer A. Elgendy, Hossam M. Tawfik

**Affiliations:** 1 Department of Endodontics, Faculty of Oral & Dental Medicine, Misr International University, Cairo, Egypt; 2 Department of Surgery, Anesthesiology and Radiology, Faculty of Veterinary Medicine, Cairo University, Giza Squa, PO Box 12211, Giza, Egypt; 3 Department of Endodontics, Faculty of Dentistry, Ain Shams University, Cairo, Egypt

**Keywords:** Biological techniques, Cell biology, Systems biology, Medical research

## Abstract

This study evaluated the biocompatibility of mineral trioxide aggregate (MTA) and Biodentine (BD) as root-end filling materials. Six mongrel dogs were divided into two equal groups according to the evaluation period; group A: one month and group B: three months. Three premolars of the same quadrant in each arch were used, summing up 36 teeth (6 teeth/dog). These teeth were randomly subdivided into three subgroups according to the root-end filling material used: MTA, BD and no root-end filling material (control). Endodontic access cavities were performed for induction of periapical pathosis. After the infection period, root canal instrumentation and obturation were accomplished. One day after root canal procedures, root-end surgery was performed. Surgical access was achieved and the root-end was resected approximately 3 mm above the apex. Root-end cavity was prepared ultrasonically and filled with the tested materials. All samples were evaluated by radiography and histopathology (Inflammation and new hard tissue formation). Data were collected and subjected to statistical analysis. In group A, MTA subgroup exhibited significant higher mean inflammatory score than BD subgroup (*P* < 0.05) while no significant difference was recorded between MTA and BD subgroups in group B (*P* > 0.05). Regarding mean mineralization score, there was no significant difference between all subgroups in both groups A and B (*P* > 0.05). Biodentine exhibited favorable biocompatibility in the initial stage of healing than MTA and comparable biomineralization.

*Clinical relevance*: Biodentine could be considered as an acceptable alternative to MTA in peri-radicular surgeries.

## Introduction

Elimination of microorganisms from the root canal system and filling of the intracanal space to avoid bacterial colonization are the main objectives of the root canal treatment (RCT)^1^. However, many factors like perforations, instrument breakage, calcifications and anatomic anomalies may fail RCT^2^. In certain circumstances, conventional RCT is not enough to treat the case and a surgical endodontic interference is needed^3^.

During peri-radicular surgeries, resection of the root-end produces an exposed apical dentin surface covered by cementum with a root canal at its center. After ultrasonic root-end preparation, root-end-filling cement is usually used to seal the root-end cavity preparation^4^. Placement of a root-end filling material after root-end resection is mandatory step to make an apical closure^5^. Furthermore, the orthograde gutta-percha filling alone is insufficient to support bone regeneration^6^.

The ideal root-end filling material must has excellent sealing ability, biocompatibility, antibacterial effect and good manipulation characteristics^7^. An ideal root-end filling material that fulfills all the required characteristics for endodontic surgery has yet to be found^8^. In the last decades, several materials like amalgam, intermediate restorative material (IRM), Super ethoxy benzoic acid (Super- EBA), glass-ionomer cement and composite resin have been applied^9^.

Mineral trioxide aggregate has less cytotoxicity, better biocompatibility and microleakage protection, giving it more clinical success over traditional root-end filling materials^10,11^. However, MTA has some drawbacks such as difficult handling, long setting time, potential discoloration, lower compressive and flexural strengths compared with those of dentin and high cost^10,12^.

Biodentine™ was introduced as a substitute to MTA. Biodentine offers similar properties to those of MTA with better consistency and faster setting time. The main components of MTA are present in Biodentine such as tricalcium silicate, calcium carbonate, and dicalcium silicate^5,12–14^.

Many in vitro studies tested MTA and BD as root-end filling materials^10^. Four studies compared the biocompatibility, two studies revealed that BD is better than MTA^15,16^ and the other two studies showed comparable results^17,18^. Nine studies compared the sealing ability, six studies showed BD to be better^19–24^, one study revealed comparable results^25^ and last two studies showed MTA to be better^5,26^.

Gray ProRoot MTA plays the leading role in the field of root-end filling^27^. To the authors’ knowledge, there is a lack of in vivo studies tested MTA and BD as root-end filling materials^8^. Therefore, this study aimed to evaluate histologically, for the first time, the biocompatibility of Gray ProRoot MTA and BD when used as root-end filling materials in a dog model. We hypothesized that both Gray ProRoot MTA and BD would have the same biocompatibility.

## Materials and methods

### Ethical approval

This work was approved by the Ethical Committee at Faculty of Dentistry, Ain Shams University, Cairo, Egypt (16‑12‑2012-Endo). All international and institutional guidelines for animal care and use were followed. The study was reported in accordance with ARRIVE guidelines.

### Animal model

The sample size was determined based on earlier studies^3,8^ using the G*power software 3.1.9.2, where a large effect size of 1.38 was detected. The significance level (α-error) was set at 0.05 and the power (1-β error) was set at 0.8 using two-sided hypothesis test. The estimated sample size was 6 for each subgroup at each evaluation period, summing up a total sample size of 36 teeth.

Six healthy 1–2-year-old male mongrel dogs (17–20 kg body weight) were selected. The animals were kept in separate kennels during the period of the study under proper conditions of nutrition, clean water, lighting, clean environment, temperature and ventilation. Dogs were provided dry food and milk before the beginning of the procedures and were shifted to soft food and milk during the procedural period.

**Figure 1:**
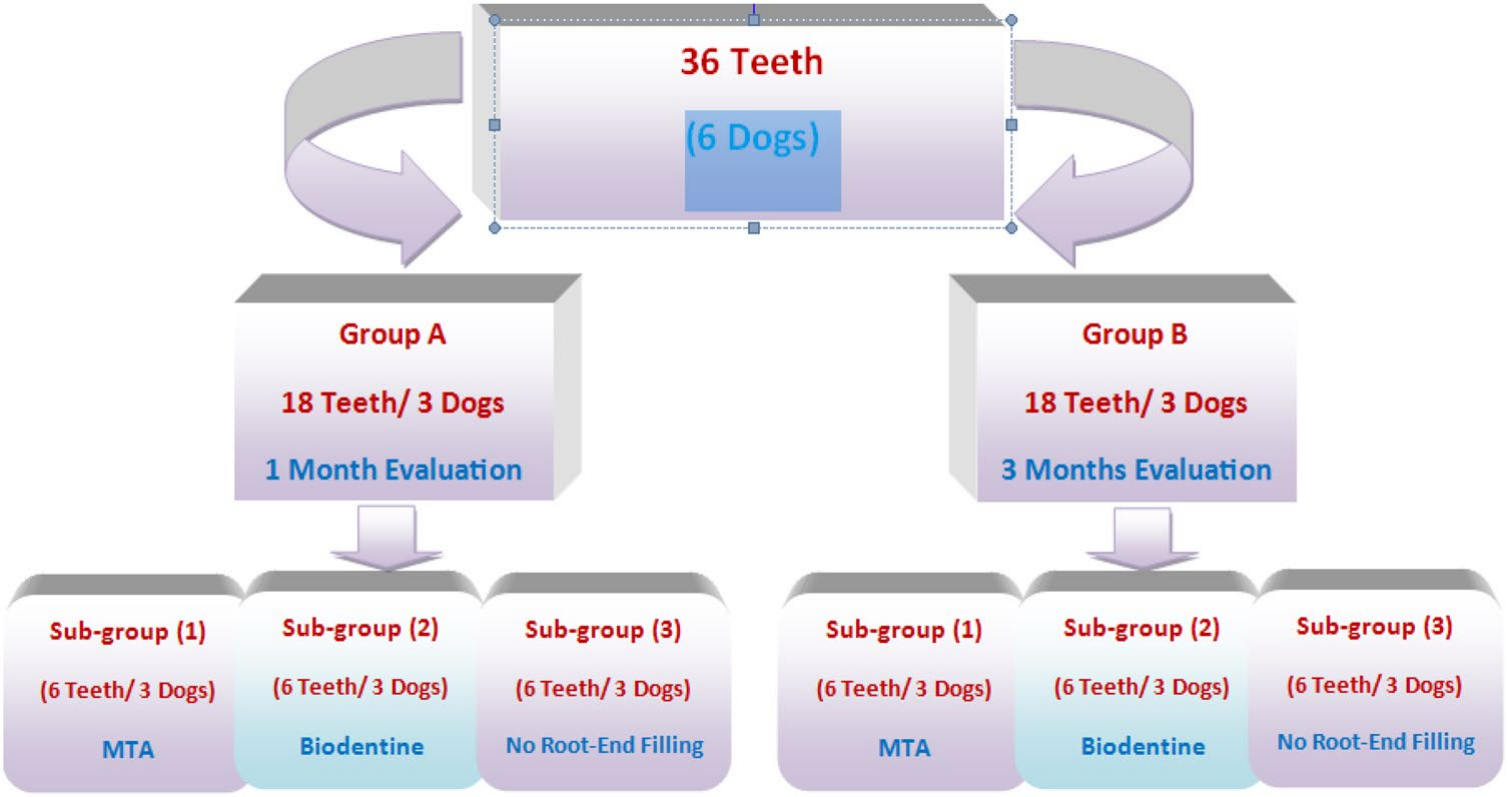
Diagram showing classification of the experimental animals and teeth in the present study.

These dogs were classified randomly into two equal groups according to the evaluation period: group A: one month and group B: 3 months. Three premolars of the same quadrant in each arch of each dog were included in this study, summing up the total number of teeth to 36 (6 premolars/ dog and 18 premolars/ group). Each group was randomly subdivided into three subgroups (6 teeth each) according to the root-end filling material used; subgroup 1 (MTA), subgroup 2 (BD) and subgroup 3: no root-end filling material (control, Fig. 1). Coded samples were used throughout the study to avoid possible bias.

### Induction of periapical pathosis

All endodontic procedures were performed under general anesthesia. The dogs were pre-medicated with 0.05 mg/kg body weight Atropine sulphate (Atropine sulphate®, ADWIA Co., Egypt) injected subcutaneously and 1mg/kg body weight Xylazine HCl (Xylaject 2%®, ADWIA Co., Egypt) injected intramuscularly. The anesthesia was induced by intravenous Ketamine HCl (Keiran®, EIMC pharmaceuticals Co., Egypt) through intravenous cannula in the cephalic vein at a dose of 5 mg/kg body weight. The anesthesia was maintained with Thiopental sodium (Thiopental sodium®, EIPICO, Egypt) at a dose of 25 mg/kg body weight 2.5% solution injected intravenous (dose to effect).

**Figure 2:**
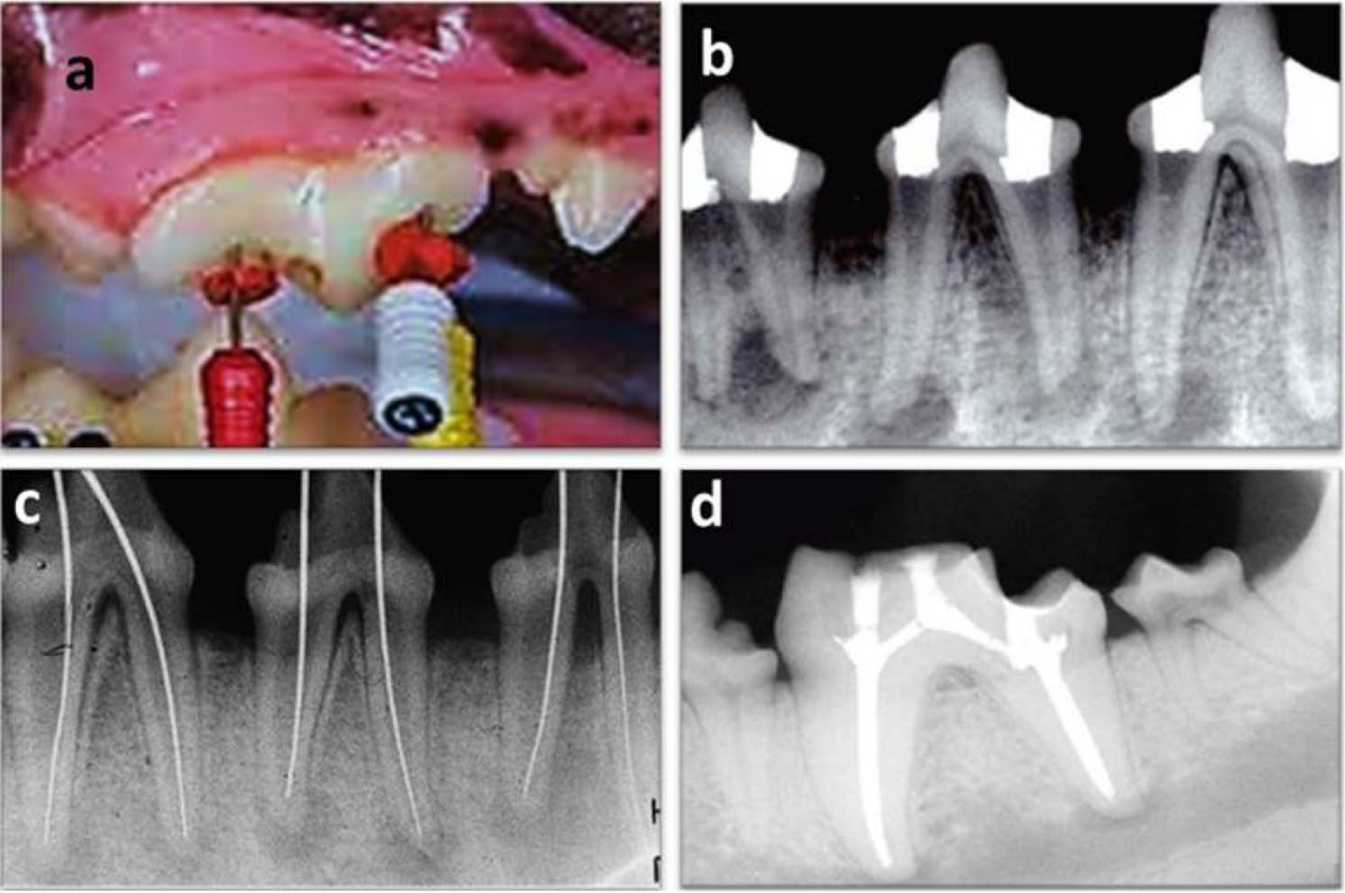
(**a**) Representative photograph showing preparation of the access cavities. (**b**) Representative photo-radiograph showing development of periapical pathosis. (**c**) Representative photo-radiograph showing determination of the working length. (**d**) Representative photo-radiograph showing obturated root canals.

Endodontic access cavities were prepared (Fig. 2a) with size #2 round bur using high speed handpiece under coolant. The pulp tissues were disrupted with sterile #15–25 Flex-o-File. Supra-gingival plaque contaminated paper points were placed into the canals and sealed with intermediate restorative material (IRM® KinderDent GmbH, Germany).

Experimental teeth were evaluated by radiography after four weeks to confirm the evidence of development of periapical pathosis (Fig. 2b). Dogs were given soft diet and pain killer, Carprofen 4.4 mg/kg (Rimadyl tab®, Zoetis, USA) orally once daily during this period.

### Root canal instrumentation and obturation

After the infection period, the previously infected experimental teeth were re-entered and the paper points were removed. Root canal length was determined by radiography using #15 & #20 Flex-o-Files (Fig. 2c). Root canal instrumentation was accomplished by using step-back technique up to a master apical file #40 Flex-o-File with conjunction with copious irrigation by 5 mL of 2.5% sodium hypochlorite followed by normal saline as final irrigant.

Root canals were subsequently dried with paper points and filled with laterally condensed gutta-percha and AH26 sealer (Fig. 2d). The access cavities were then sealed with intermediate restorative material.

### Surgical procedures

One day after root canal procedures, peri-radicular surgery was performed by a single operator (MN). This procedure was performed on two premolars in each quadrant while the third one did not undergo root-end surgery (Control).

Surgical access was achieved by a full muco-periosteal flap with two releasing incisions using a scalpel blade size #15 (BD, São Paulo, São Paulo, Brazil). The flap was reflected and the root apex was approximately localized via pre-determined root canal length. The covering cortical bone of the root ends was removed to expose the required apex of the tooth.

Apical curettage was performed using lucas curette size #85 and #86 (Hu-Friedy, Rio de Janeiro, Rio de Janeiro, Brazil) to remove all necrotic tissues and bone particles from the peri-radicular area. During the surgical phase, 0.5 mL of 2% Xylocaine HCl with 1:50,000 epinephrine was injected into the surgical site to achieve maximum hemostasis.

The root-end was resected approximately 3 mm above the apex using a carbide bur mounted on a high speed handpiece under water coolant at a 90° angle to the long axis of the root. The root-end resection was not performed under microscope magnification. Then, 3 mm in depth from the resected surface was prepared in each sample using an ultrasonic tip (Ultrasonic tip, E32D NSK, Tochigi, Japan) powered by an ultrasonic device (Piezon Master, EMS, Nyon, Switzerland( at a frequency of 32 kHz. Intermittent pressure was employed within-and-out motion to start preparation, then the depth was increased to 3 mm from the resected surface. Finally the tip was moved circumferentially to complete the preparation. A periodontal probe served as measuring device for preparation depth. Then the root-end cavity was irrigated with sterile normal saline.

Root-end cavity preparations were dried with paper points and filled with the tested materials which were mixed according to the previously described manufacturer’s recommendations. Using the carrier (Ultrasonic tip, E32D NSK, Tochigi, Japan), the material was dispensed into the root-end cavity and compacted using a small plugger (Dentsply, York, PA). Excess material was removed and the surface of the root was cleaned with moist gauze. Placement of the tested materials was confirmed by radiography.

The muco-periosteal flap was repositioned and fixed with moderated digital pressure and moist gauze. Suturing with silk thread 4/0 (Ethicon Johnson, São Paulo, São Paulo, Brazil) was done. Sutures were removed 7 days after surgery. Dogs were given soft diet and pain killer as mentioned before during this period.

## Methods of evaluation

### Radiography evaluation

Periapical radiographs taken after induction of the periapical lesion were compared with follow-up radiographs taken at one month (Group A) and three months (Group B).

Radiography evaluation was done using a modification for the scoring system established by Molven et al.^28^ as follows:

Score 0: no healing (Increase in size of former radiolucency), Score 1: unsatisfactory healing (No reduction of former radiolucency), Score 2: uncertain healing (Some reduction of former radiolucency), Score 3: incomplete healing and Score 4: complete healing.

The radiographs were evaluated independently by 2 examiners (AMA and AAE). A specific healing category was selected when the two examiners had the same judgment.

### Histopathology evaluation

Animals were sacrificed by overdose of general anesthetic (Thiopental sodium rapidly intravenous). Jaws were resected and bone segments including the teeth were cut and prepared for histopathological evaluation. The remnant of the animal body was handled in a proper way (cremated).

Obtained bone blocks were fixed in 10% buffered formalin solution with ratio 1:50. After two weeks of fixation, blocks were decalcified using 17% EDTA solution. The decalcifying solution was renewed on daily basis for about 120 days. After decalcification, samples were dehydrated in ascending concentrations of ethanol and then embedded in paraffin blocks. Blocks were sectioned in bucco-lingual sections at 6µm thickness. Sections were stained with hematoxylin and eosin dye and evaluated by an experienced oral pathologist blinded to the experimental groups. The evaluation included both quantitative and qualitative assessments as follows:

#### Quantitative evaluation

Inflammatory tissue reaction at the periapical area was evaluated using a scoring system according to Huang et al.^29^ as follows: Score 0: no inflammatory tissue infiltration (No inflammatory cells or edema detected), Score 1: mild inflammatory tissue infiltration (Sparse infiltration of inflammatory cells with infrequent edema formation), Score 2: moderate inflammatory tissue infiltration (Moderate infiltration of inflammatory cells with frequent edema formation) and Score 3: severe inflammatory tissue infiltration (Dense infiltration of inflammatory cells with intense edema formation).

New hard tissue formation was also assessed using a scoring system according to Huang et al.^29^ as follows: Score 0: absence of new hard tissue formation, Score 1: partial formation of new hard tissues and Score 2: complete formation of new hard tissue.

### Qualitative evaluation

Stained sections were examined under a light microscope at magnification X100, X200 and X400 for assessment of the periapical area, detection of the inflammatory nature and presence of new hard tissue formation.

All measurements were performed by two calibrated and blinded examiners in two different sessions.

### Data collection and statistical analysis

Collected data were represented as the mean and standard deviation (SD) values. All data were in form of scores, so non-parametric tests were used for the comparisons. Mann–Whitney U test was used to compare between two groups. Kruskal–Wallis test was used to compare between more than two groups. Wilcoxon signed-rank test was used to study the effect of time in comparisons with two follow up times. Dunn's test was used for pair-wise comparisons when Kruskal–Wallis test had significant results. The significance level was set at *P* ≤ 0.05. Statistical analysis was performed with SPSS Statistics Version 20 for Windows (SPSS®, Inc., IBM Company, USA).

## Results

### Radiography findings

#### Effect of root-end filling materials on radiographic periapical healing score

**Figure 3:**
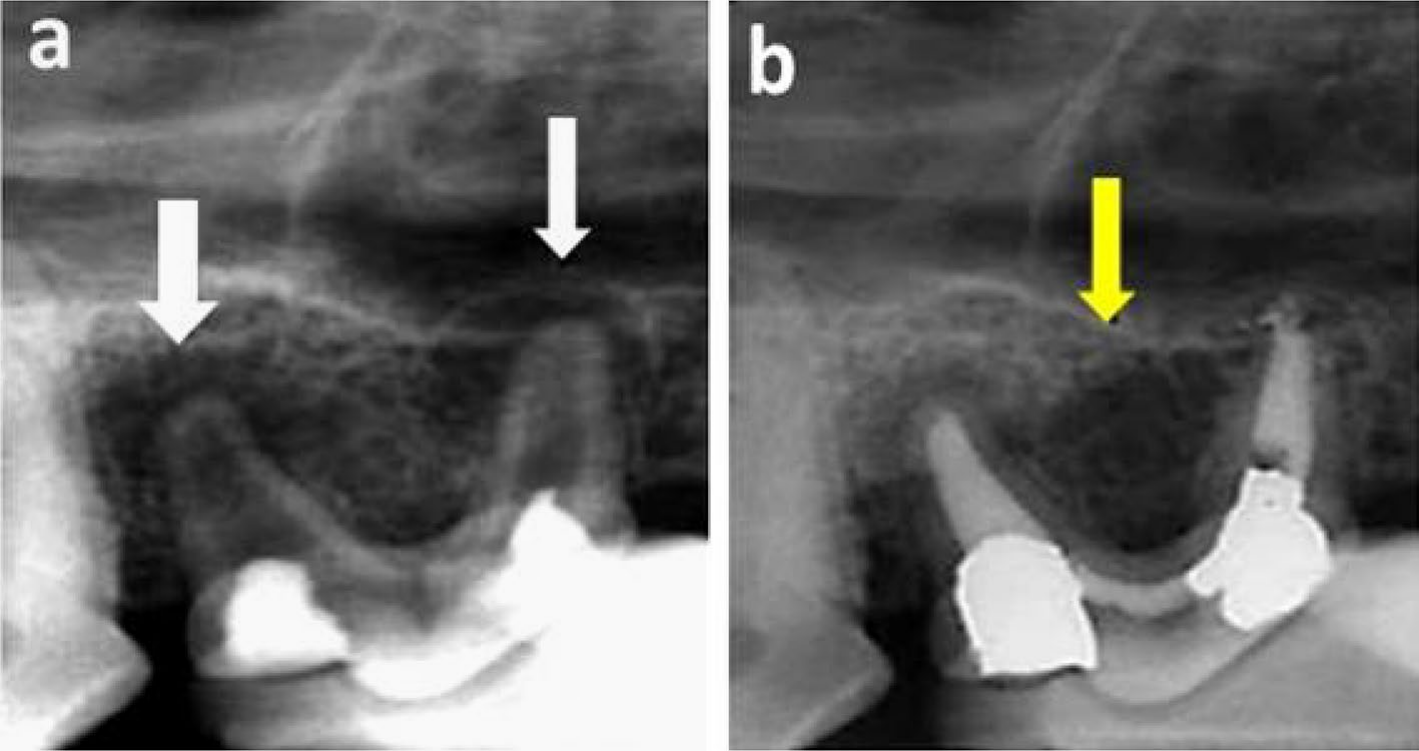
(**a**) Representative pre-operative photo-radiograph confirming presence of apical radiolucency (white arrows). (**b**) Representative post-operative photo-radiograph of a sample in subgroup A3 (Control subgroup after one month) showing incomplete healing (yellow arrow, score 3).

**Figure 4:**
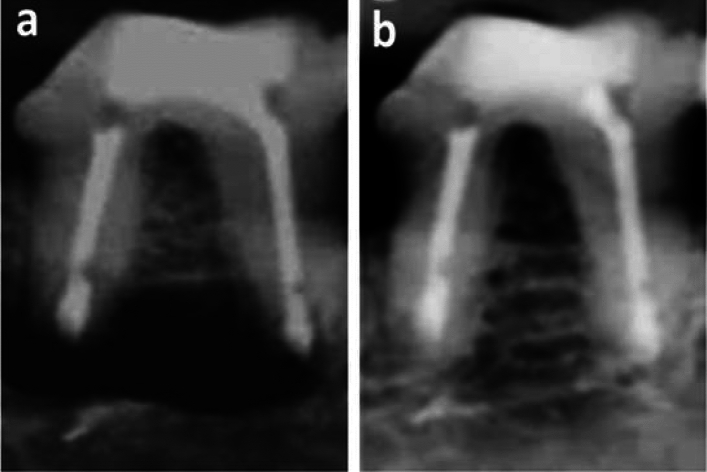
(**a**) Representative pre-operative photo-radiograph confirming presence of a large apical radiolucency. (**b**) Representative post-operative photo-radiograph of subgroup A2 (BD after one month) showing incomplete healing (score 3).

In group A (After one month), the mean radiography healing scores in subgroup 3 (Control), subgroup 2 (BD) and subgroup 1 (MTA) were 3.17 ± 0.98, 1.50 ± 0.84 and 1.00 ± 0.63, respectively (Figs. 3 and 4). Statistically, there was a significant difference between the three subgroups (*P* = 0.006). Pair-wise comparisons revealed that subgroup 3 (Control) showed the statistically significant highest mean radiography healing score. There was no statistically significant difference between subgroup 1(MTA) and subgroup 2 (BD); both showed statistically significant lower mean radiography healing score than subgroup 3 (Control).

**Figure 5:**
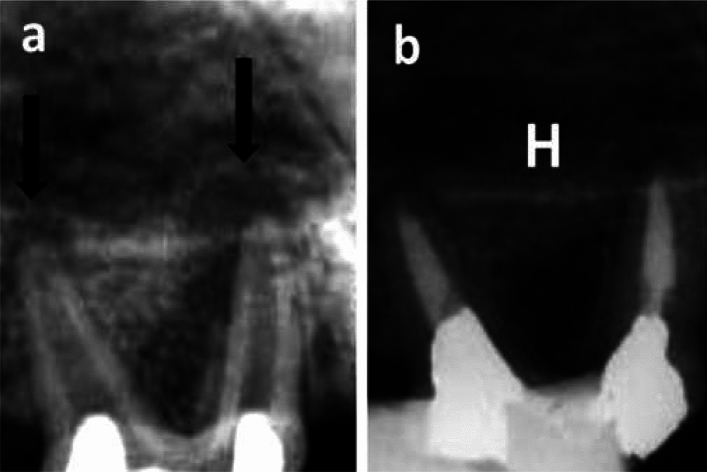
(**a**) Representative pre-operative photo-radiograph confirming presence of apical radiolucency (black arrows). (**b**) Representative post-operative photo-radiograph of subgroup B1 (MTA after three months) showing complete healing (H, score 4).

In group B (After 3 months), the lowest mean radiography healing score was demonstrated in subgroup 2, reaching 2.17 ± 0.41 while the highest mean radiography healing score was demonstrated in subgroup 3, reaching 3.67 ± 0.52 as shown in (Table 1). The mean radiograph**y** healing score in subgroup 1 was intermediate (2.83 ± 0.75, Fig. 5). Statistically, there was a significant difference between the three subgroups (*P* = 0.007). Pair-wise comparisons revealed that subgroup 3 showed the highest mean radiography healing score with non-statistically significant difference from subgroup 1(MTA) but a statistically significant higher mean radiography healing score than subgroup 2 (BD) was recorded. Sub-group 2 (BD) showed the lowest mean radiography healing score with no statistically significant difference from subgroup 1 (*P* > 0.05) and a statistically significant difference from subgroup 3 (*P* < 0.05).

**Table 1:** Mean and standard deviation (SD) for radiography healing scores in all groups and subgroups.

Groups	Subgroup (1) MTA		Subgroup (2) Biodentine		Subgroup (3) no filling (control)		*P* value
	Mean	SD	Mean	SD	Mean	SD	
Group A (1 month)	1.00^B^	0.63	1.50^B^	0.84	3.17^A^	0.98	0.006*
Group B (3 months)	2.83^AB^	0.75	2.17^B^	0.41	3.67^A^	0.52	0.007*

*Significant at *P* ≤ 0.05, Different superscripts in the same row are statistically significantly different.

### Effect of time intervals on radiography periapical healing score for each subgroup

In subgroup 1 (MTA), the mean radiography healing score after one month evaluation period was 1.00 ± 0.63. An increase in this score was recognized after 3 months evaluation period (2.83 ± 0.75). Statistically, this increase was significant (*P* = 0.020).

In subgroup 2 (BD), the mean radiography healing score after one month evaluation period was 1.50 ± 0.84. An increase in this score was reported after 3 months evaluation period (2.17 ± 0.41). Statistically, this increase was not significant (*P* = 0.102).

In subgroup 3 (Control), the mean radiography healing score after one month evaluation period was 3.17 ± 0.98. An increase in this score was recorded after 3 months evaluation period (3.67 ± 0.52). Statistically, this increase was not significant (*P* = 0.180).

### Histopathology findings

### Quantitative findings

#### Effect of root-end filling materials on inflammatory tissue reaction at the periapical area in both groups

In group A, the mean inflammatory scores in subgroup 3 (Control), subgroup 2 (BD) and subgroup 1 (MTA) were 1.33 ± 0.52, 2.17 ± 0.41 and 2.83 ± 0.41, respectively (Table 2). Statistically, there was a significant difference between the three subgroups (*P* = 0.003). Pair-wise comparisons revealed that subgroup 1 showed the statistically significant highest mean inflammatory score. Subgroup 2 showed statistically significant lower mean inflammatory score than subgroup 1. Subgroup 3 showed the statistically significant lowest mean inflammatory score.

**Table 2:** Mean inflammatory scores and standard deviation (SD) in all groups and subgroups.

Groups	Subgroup (1) MTA		Subgroup (2) Biodentine		Subgroup (3) no filling (control)		*P* value
	Mean	SD	Mean	SD	Mean	SD	
Group A 1 month	2.83^A^	0.41	2.17^B^	0.41	1.33^C^	0.52	0.003*
Group B 3 months	0.83^AB^	0.41	1.33^A^	0.52	0.33^B^	0.52	0.019*

*Significant at *P* ≤ 0.05, Different superscripts in the same row are statistically significantly different.

In group B, the mean inflammatory scores in subgroup 3 (Control), subgroup 1 (MTA) and subgroup 2 (BD) were 0.33 ± 0.52, 0.83 ± 0.41and 1.33 ± 0.52, respectively as shown in (Table 2). Statistically, there was a statistically significant difference between the three subgroups (*P* = 0.019). Pair-wise comparisons revealed that subgroup 2 (BD) showed the highest mean inflammatory score with no statistically significant difference from subgroup 1 (*P* > 0.05) but a statistically significant higher inflammatory score than subgroup 3 (*P* < 0.05) was noticed. Subgroup 3 showed the lowest mean inflammatory score with no statistically significant difference from subgroup 1 (*P* > 0.05) and a statistically significant difference from subgroup 2 (*P* < 0.05).

### Effect of time intervals on inflammatory tissue reaction at the periapical area in different subgroups

In subgroup 1 (MTA), the mean inflammatory score after one month evaluation period was 2.83 ± 0.41. A decrease in this score was recognized after 3 months evaluation period (0.83 ± 0.41). Statistically, this decrease was significant (*P* = 0.024).

In subgroup 2 (BD), the mean inflammatory score after one month evaluation period was 2.17 ± 0.41. A decrease in this score was recorded after 3 months evaluation period (1.33 ± 0.52). Statistically, this decrease was significant (*P* = 0.025).

In subgroup 3 (Control), the mean inflammatory score after one month evaluation period was 1.33 ± 0.52. A decrease in this score was reported after 3 months evaluation period (0.33 ± 0.52). Statistically, this decrease was significant (*P* = 0.034).

### New hard tissue formation

#### Effect of root-end filling materials on the rate of new hard tissue formation in both groups

In group A, the mean mineralization scores in subgroup 3 (Control), subgroup 2 (BD) and subgroup 1 (MTA) were 1.17 ± 0.98, 0.83 ± 0.41 and 0.17 ± 0.41, respectively. Statistically, there was no significant difference between the three subgroups (*P* = 0.067).

In group B, the mean mineralization scores in subgroup 3 (Control), subgroup 1 (MTA) and subgroup 2 (BD) were 2.17 ± 0.98, 1.83 ± 0.75 and 1.17 ± 0.41, respectively as shown in (Table 3). Statistically, there was no significant difference between the three subgroups (*P* = 0.067).

**Table 3:** Mean mineralization scores and standard deviation (SD) in all groups and subgroups.

Groups	Subgroup (1) MTA		Subgroup (2) Biodentine		Subgroup (3) no filling (control)		*P* value
	Mean	SD	Mean	SD	Mean	SD	
Group A 1 month	0.17	0.41	0.83	0.41	1.17	0.98	0.067
Group B 3 months	1.83	0.75	1.17	0.41	2.17	0.98	0.109

*Significant at *P* ≤ 0.05.

#### Effect of time intervals on the rate of new hard tissue formation in different subgroups

In subgroup 1 (MTA), the mean mineralization score after one month evaluation period was 0.17 ± 0.41. An increase in this score was recognized after 3 months evaluation period (1.83 ± 0.75). Statistically, this increase was significant (*P* = 0.023).

In subgroup 2 (BD), the mean mineralization score after one month evaluation period was 0.83 ± 0.41. An increase in this score was recorded after 3 months evaluation period (1.17 ± 0.41). Statistically, this increase was significant (*P* = 0.014).

In subgroup 3 (Control), the mean mineralization score after one month evaluation period was 1.17 ± 0.98. An increase in this score was reported after 3 months evaluation period (2.17 ± 0.98). Statistically, this increase was significant (*P* = 0.014).

### Qualitative findings in different subgroups

### Subgroup 1 (MTA)

**Figure 6:**
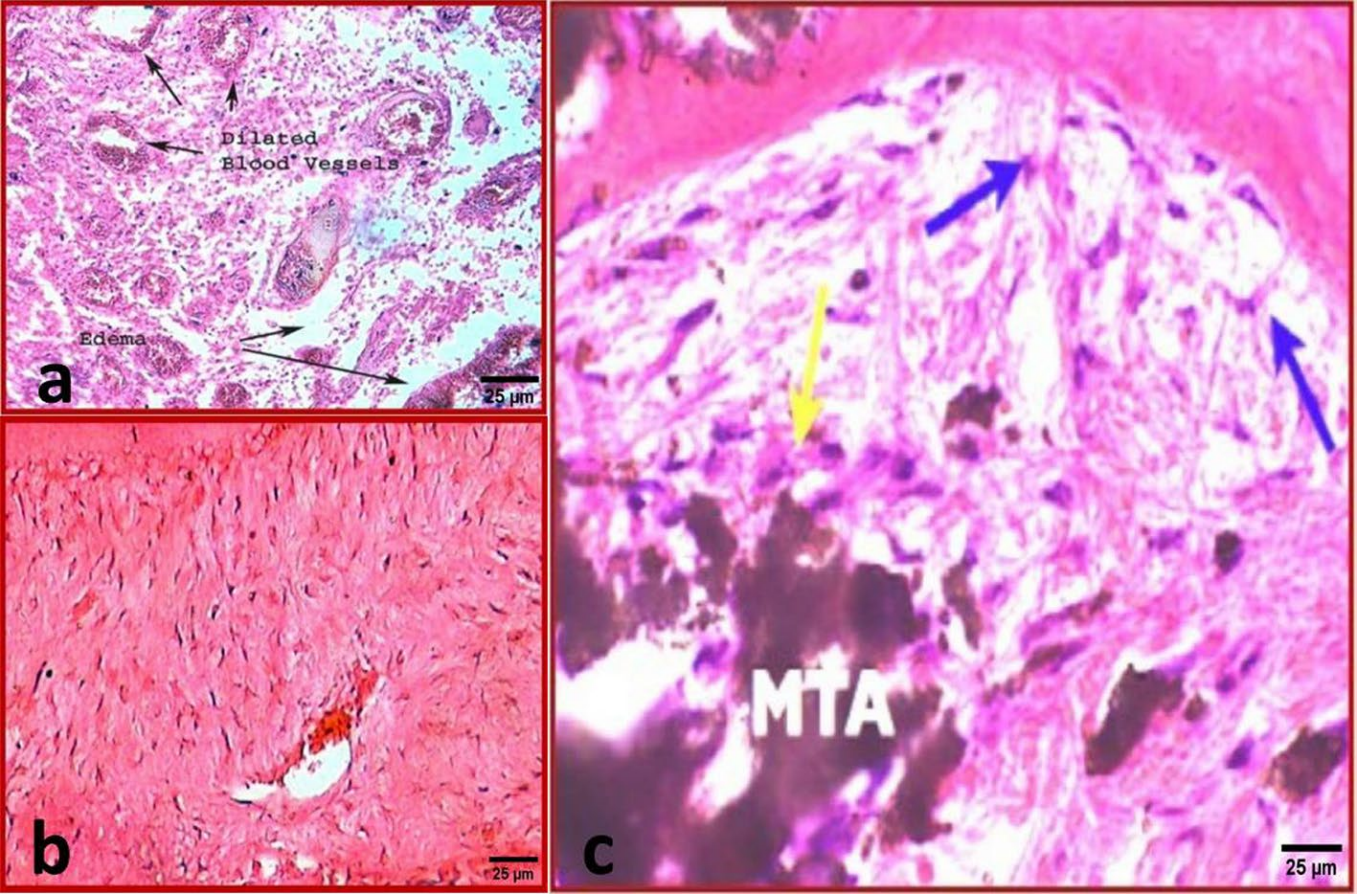
(**a**) Representative photomicrograph of subgroup A1 (MTA after one month) showing dense inflammatory cells infiltration, numerous dilated blood vessels and edema spaces (Score 3). (X400, H&E). (**b**) Representative photomicrograph of subgroup B1 (MTA after three months) showing mild inflammatory cells infiltration (Score 1). (X400, H&E). (**c**) Representative photomicrograph of subgroup B1showing no inflammatory cells infiltration (Score 0) around MTA in the periapical area (Yellow arrow). Notice the mineralized tissue formed on the surface of MTA (Blue arrows). (X400, H&E).

In group A (One month), dense inflammatory cells infiltration (Score 3) was seen with numerous dilated blood vessels and edema spaces engorged by decomposed RBCs. No evidence of newly deposited hard tissue (Fig. 6a).

In group B (Three months), mild chronic inflammatory cells infiltration (Score 1) was observed and separated islands of lymphocytes, macrophages and plasma cells were evident and interlaced with new fibrous ligaments (Fig. 6b). Mineralized tissue was formed on the surface of MTA (Fig. 6c).

### Subgroup 2 (BD)

**Figure 7:**
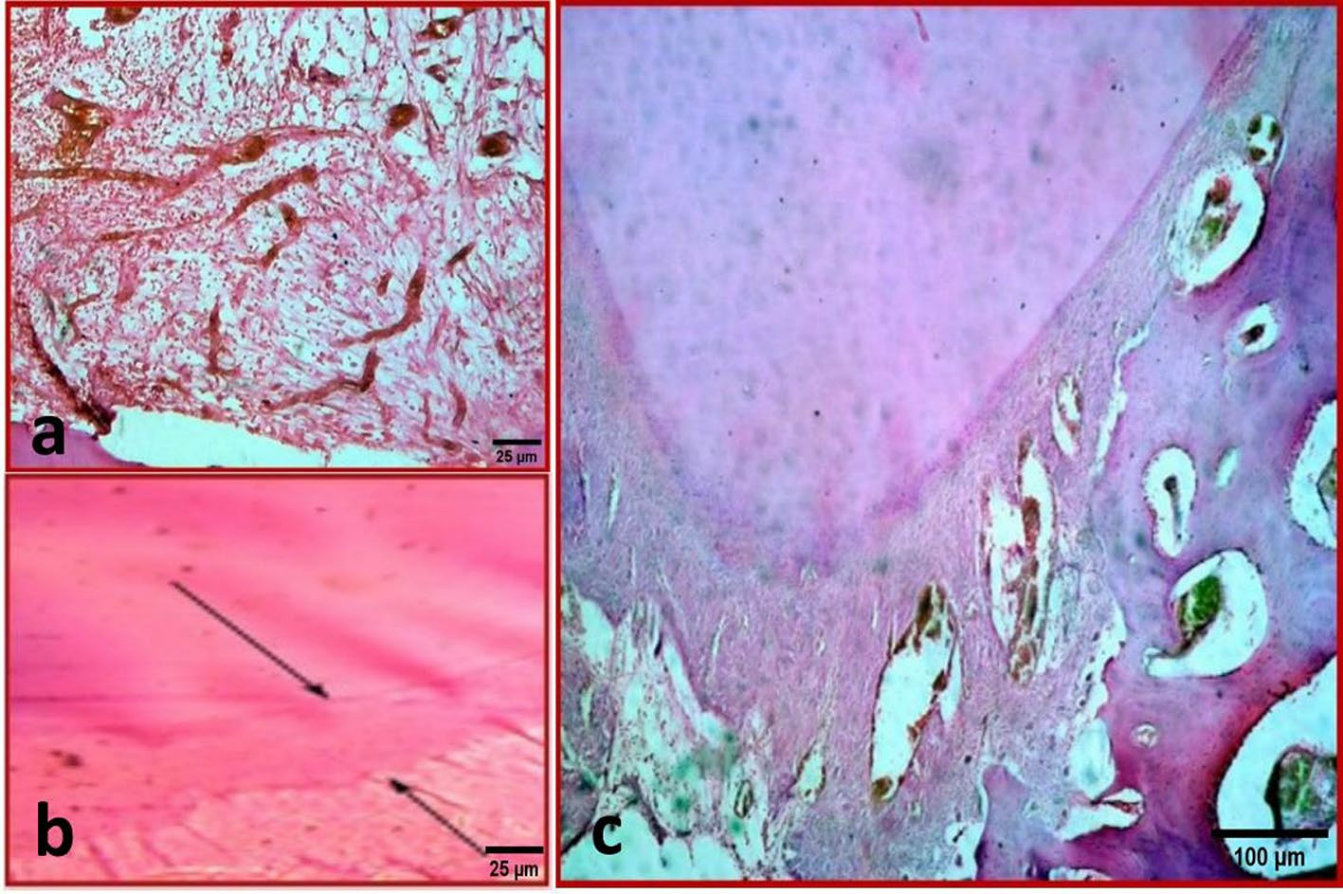
(**a**) Representative photomicrograph of subgroup A2 (BD after one month) showing moderate inflammatory cells infiltration (Score 2). (X400, H&E). (**b**) Representative photomicrograph of the same subgroup showing newly deposited hard tissue (Black arrows) (Score 1). (X400, H&E). (**c**) Representative photomicrograph of subgroup B2 (BD after three months) showing noticeable increase in blood vessels. (X100, H&E).

In group A, moderate inflammatory cells infiltration (Score 2) was seen with dilated blood vessels and edema spaces (Fig. 7a). New osseous-like tissue formation could be recognized that was demarcated by dark lines (Fig. 7b).

In group B, the former appearance of chronic inflammatory cells infiltration decreased but was still evident with numerous dilated blood vessels and edema spaces engorged by decomposed RBCs (Fig. 7c). New osseous-like tissue formation was evident which was demarcated by dark lines.

### Subgroup 3 (Control)

In group A, mild inflammatory cells infiltration (Score 1) was seen. New osseous-like tissue formation could be recognized that was demarcated by dark lines.

**Figure 8:**
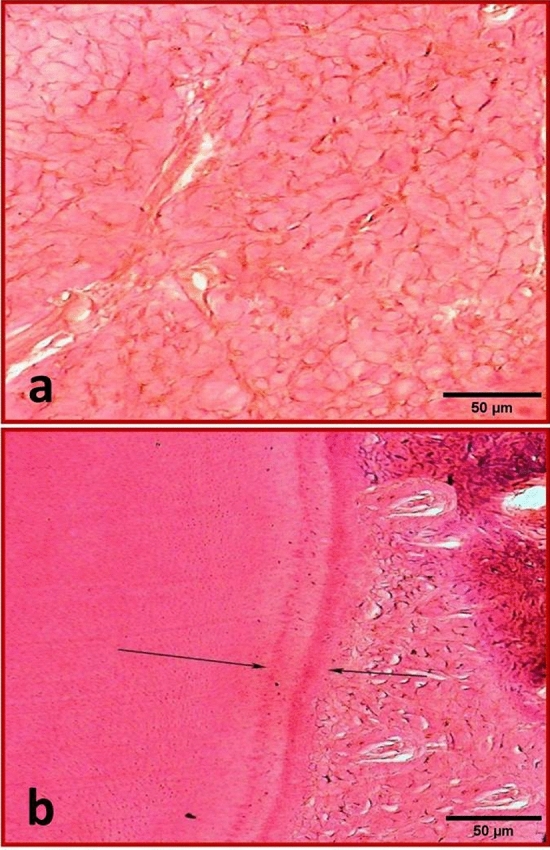
(**a**) Representative photomicrograph of subgroup B3 (Control subgroup after three months) showing no inflammatory cell infiltration (Score 0). (X200, H&E). (**b**) Representative photomicrograph of the same subgroup showing newly deposited osseous-like tissue (Black arrows, score 2). (X200, H&E).

In group B, no inflammatory cells infiltration (Score 0) was seen (Fig. 8a). New osseous-like tissue formation could be recognized that was demarcated by dark lines. The new osseous-like tissue showed the traditional Howship’s lacunae filled with osteocytes (Fig. 8b).

## Discussion

Complete obliteration of the root canal system and induction of a fluid tight seal play a crucial role in successful endodontic therapy. Root-end resection and root-end filling are common surgical procedures for endodontic treatment^20,21^.

This study evaluated the biocompatibility of two different root-end filling materials, Gray ProRoot MTA and BD. The two tested root-end filling materials were selected because the manufacturer of each material claims its superior qualities in clinical performance. The present results revealed that BD exhibits favorable biocompatibility in the initial stage of healing than MTA and comparable biomineralization. Therefore, the hypothesis of this study is accepted.

The choice of dog as an animal model is based on the fact that dogs have similar apical repair compared with humans in shorter duration (average one sixth of human) due to higher growth rate^11,12,30^. Double-rooted premolars in one quadrant in each arch were selected summing up a total of 6 teeth in each dog increasing the whole number of samples for a reliable statistical analysis^8^. Premolars are accessible for endodontic procedures, also having average-sized canals for endodontic manipulation^31,32^. Age range selected was 1–2 years which was suitable for this study because the premolars are mature at this age range and the animal can withstand general anesthesia procedure at multiple interventions.

Samples were divided into 2 groups according to observation period; one month and three months. One month interval was selected for evaluation of short-term reaction to the committed treatment. Three months interval was selected for evaluation of long-term reaction. This is in agreement with a previous in vivo study in which the periapical healing of both was evaluated by radiography and histopathology after 1 month and 3 months^3^.

Induction of periapical infection was done in order to simulate clinical conditions. The contaminated paper points were left inside the root canals for four weeks in order to leave sufficient duration for establishment of periapical pathosis as mentioned before by earlier authors^32,33^.

Although root-end resection is a mandatory step in endodontic surgery, it reduces the total root length and supported root surface. Therefore it changes the biomechanical response of the tooth that may result in unfavorable stress distribution and may increase tooth mobility^34^. Nevertheless, a 3 mm root-end resection appeared to be mechanically acceptable in order to ensure the long-term prognosis of endodontic surgery^35^. Moreover, increasing the depth of the retrograde filling significantly decreased apical leakage; there was also a significant increase in leakage as the amount of bevel increased^34^.

Apical ramifications and laterals canals are very common near root tip^36^. So, resection at the depth of 3 mm was done. This is in agreement with earlier studies^34–36^. The angle of cutting was 90° to avoid exposing more patent dentinal tubules to bacterial contamination^37^. The microleakage increases significantly with increased angulations of the resected root-end^34,37^. On the other hand, the results of an in vitro study showed that when an adequate retrograde cavity depth is prepared, variation in the root-end cutting angle does not necessarily cause any difference in microleakage^36^.

In group A, most of samples in MTA and BD subgroups showed severe to moderate inflammation. Subgroup 1 exhibited more inflammatory infiltration than subgroup 2 (BD) samples but without significant difference. These results are in accordance with results of other studies that showed comparable healing of BD used in root-end surgery in comparison with MTA^38–40^. These findings are logically attributed to the immediate inflammatory reaction of the peri-radicular tissues to the performed surgical treatment protocol superimposed by the immunological reaction against the previously induced infection. On contrary, the mean inflammatory score (Mild to moderate) in subgroup 3 (Control) was significantly lower than other subgroups. This may be attributed to absence of root-end surgery in subgroup 3.

Regarding new hard tissue formation in group A, the results showed a variable behavior among all the experimental subgroups. The lowest mean mineralization score was demonstrated by samples of MTA while the highest score was demonstrated by the control teeth followed by samples of BD. A possible explanation for superiority of BD is its bioactivity that activates angiogenesis and progenitor periodontal cells, thus promoting healing and remineralization^40^. Moreover, BD lacks cytotoxicity and stimulates collagen fiber and fibroblast formation^41^. Biodentine also stimulates osteogenic differentiation of human bone marrow stem cells^39^. Lee et al. suggested use of BD and MTA as root-end filling materials because in contact with mesenchymal stem cells they induce osteoblast differentiation^38^.

In group B, the inflammatory scores decreased in all subgroups compared to those at one month evaluation period. This may be attributed to subsiding of inflammation by time.

Regarding new hard tissue formation, healing took place in all surgical sites after three months. The highest mean mineralization score was demonstrated in subgroup 3. There was no significant difference in mean mineralization score between MTA and BD subgroup. These results are in agreement with results of other studies that showed comparable healing of BD and MTA when used in root-end surgery^40,42,43^. A possible explanation for biocompatibility of MTA after three months evaluation is its unique feature that is osseous-like tissue formation directly on its surface^44^. It is possible that calcium oxide, in MTA formulation, reacts with water or tissue fluids, forming calcium hydroxide and stimulating hard tissue deposition^5,45^.

In the present study, mineralized tissue was also seen on the surfaces of MTA and BD and the resected dentin. However, the cementum and bone have very similar characteristics, and there was no specific staining technique to differentiate between the two structures. Hence, the term osseous-like tissue was used, mainly on the basis of the location of this mineralized tissue and some features observed on hematoxylin–eosin stained slides. The difficulty in distinguishing between cementum and bone in the staining is considered a challenge associated with the qualitative assessment. Therefore, the nature of this mineralized tissue and mechanism of its formation need further investigation in the future.

In the present work, the histology findings are in agreement with the radiography findings. Similar findings were recorded before after peri-radicular surgery^3^. However, periapical radiography could not detect the difference. It can be inferred that minute differences such as reformation of PDL, cementum, and quality of bone cannot always be observed on periapical radiographs, even though the success criteria were based on the correlation between histologic findings and radiographic features as mentioned before^3,28^.

There are some limitations in this study such as the limited number of animals and assessment of healing only at 2 time points after surgery. Hence, the healing dynamic associated with the tested materials is unknown. The differences between MTA and BD might be more dramatic or non-significant at an earlier or extended follow-up period. Furthermore, bacteria profiles present in the canal may not be as complex as most clinical situations. Therefore, caution must be taken in directly applying these results in clinical conditions. Therefore, future studies are recommended, especially regarding an extended follow-up period and more complex bacterial profiles.

## Conclusion

Biodentine exhibited favorable biocompatibility in the initial stage of healing than MTA and comparable biomineralization. Therefore, BD could be considered as an acceptable alternative to MTA in peri-radicular surgeries.

## Data Availability

The datasets used and/or analyzed during the current study are available from the corresponding author on reasonable request.
